# Anemia and iron deficiency in COPD patients: prevalence and the effects of correction of the anemia with erythropoiesis stimulating agents and intravenous iron

**DOI:** 10.1186/1471-2466-14-24

**Published:** 2014-02-24

**Authors:** Donald S Silverberg, Ram Mor, Melanie Tia Weu, Doron Schwartz, Idit F Schwartz, Gil Chernin

**Affiliations:** 1 Nephrology Department, Tel-Aviv Sourasky Medical Center and Sackler Faculty of Medicine, Tel-Aviv University, Tel-Aviv, Israel; 2 Pulmonology Institute, Tel-Aviv Sourasky Medical Center and Sackler Faculty of Medicine, Tel-Aviv University, Tel-Aviv, Israel; 3 Department of Nephrology, CHU de Yopougon Hospital, Abidjan, Ivory Coast

**Keywords:** Iron deficiency, Anemia, COPD, Renal failure, Erythropoietin

## Abstract

**Background:**

Little is known about iron deficiency (ID) and anemia in Chronic Obstructive Pulmonary Disease (COPD). The purposes of this study were: (i) To study the prevalence and treatment of anemia and ID in patients hospitalized with an exacerbation of COPD. (ii) to study the hematological responses and degree of dyspnea before and after correction of anemia with subcutaneous Erythropoiesis Stimulating Agents (ESAs) and intravenous (IV) iron therapy, in ambulatory anemic patients with both COPD and chronic kidney disease.

**Methods:**

(i) We examined the hospital records of all patients with an acute exacerbation of COPD (AECOPD) to assess the investigation, prevalence, and treatment of anemia and ID. (ii) We treated 12 anemic COPD outpatients with the combination of ESAs and IV-iron, given once weekly for 5 weeks. One week later we measured the hematological response and the severity of dyspnea by Visual Analogue Scale (VAS).

**Results:**

(i) Anemia and iron deficiency in hospitalized COPD patients: Of 107 consecutive patients hospitalized with an AECOPD, 47 (43.9%) were found to be anemic on admission. Two (3.3%) of the 60 non-anemic patients and 18 (38.3%) of the 47 anemic patients had serum iron, percent transferrin saturation (%Tsat) and serum ferritin measured. All 18 (100%) anemic patients had ID, yet none had oral or IV iron subscribed before or during hospitalization, or at discharge. (ii) Intervention outpatient study: ID was found in 11 (91.7%) of the 12 anemic ambulatory patients. Hemoglobin (Hb), Hematocrit (Hct) and the VAS scale scores increased significantly with the ESAs and IV-iron treatment. There was a highly significant correlation between the ∆Hb and ∆VAS; r_s_ = 0.71 p = 0.009 and between the ∆Hct and ∆VAS; r_s_ = 0.8 p = 0.0014.

**Conclusions:**

ID is common in COPD patients but is rarely looked for or treated. Yet correction of the ID in COPD patients with ESAs and IV iron can improve the anemia, the ID, and may improve the dyspnea.

## Background

Anemia is seen in 10-30% of Chronic Obstructive Pulmonary Disease (COPD) patients [1-15]. The anemia is associated with increased mortality and morbidity including increased hospitalization and increased health care costs [1-15]. The anemia in COPD has been associated with a negative effect on dyspnea and walking distance [5], on circulatory efficiency (as compared to non-anemic COPD patients) as judged by lower peak oxygen uptake and lower peak work rate [16] and with the need for home oxygen therapy and lower mean peripheral oxygen values both at rest and during exercise [9] Although there are many contributors to the anemia it is probably caused mainly by the inflammatory nature of COPD [12], with the increased cytokine production causing anemia and iron deficiency (ID) [17,18].

Little information exists on the role of iron deficiency (ID) in COPD although serum iron and %TSat levels were found in a US population-based cross sectional study to be directly related to one second Forced Expiratory Volume (FEV 1) levels [19]. In addition iron intake in COPD patients has been found to be about half that of normal controls [20].

Surprisingly, not a single study has, to our knowledge, been reported using either of these two agents, Erythropoiesis Stimulating Agents (ESAs) or intravenous (IV) iron, in the treatment of the anemia and/or iron deficiency of COPD.

Our hypothesis was that functional ID may be a prevalent condition in patients with COPD. To test this hypothesis, we reviewed retrospectively a series of 107 patients admitted for AECOPD.

In our medical clinic devoted to the treatment of anemia due to chronic kidney disease (CKD) and congestive heart failure (CHF) [21,22], we found 12 patients that were also suffering from COPD. We evaluated the clinical effect of treating anemia and ID with ESAs and IV iron.

## Methods

Approval of the study was granted by the Ethical Committee of the Tel-Aviv Medical Center.

### Study 1

We examined the hospital records of all patients admitted with an AECOPD between January 1, 2012 to December 31 2012 to assess the prevalence, the investigation and treatment of anemia and ID in these patients. COPD diagnosis was based on information encoded in the administrative database.

### Study 2

We identified 12 anemic (defined as Hb < 12 g/dl) patients with an established diagnosis of COPD who had been treated in our clinic. We analyzed the effects of the combination of subcutaneous ESAs and IV-iron on dyspnea in these patients. Patient characteristics that were examined at baseline included presence of CKD, CHF, hypertension, diabetes, dyslipidemia and the medications taken.

The anemia was corrected with the combination of ESAs and IV-iron given once weekly for 5 weeks and the final assessment was done one week after the last dose. IV iron (200 mg of elemental iron) was given as two 5 ml ampoules- each containing a total of 100 mg of elemental iron) of Venofer -Ferric Sucrose (Vifor Int, St. Galen, Switzerland). The doses were given one week apart (a total of 1000 mg elemental iron) for 5 weeks. ESAs were given weekly in the form of 10,000 International Units (IU) of subcutaneous (sc) Recormon (Roche, Basel, Switzerland) for five weeks at the same time that the IV iron was given. Weight, height, and blood pressure were measured on every weekly visit. Blood work, done initially, at every visit and one week after the fifth treatment, included: Hemoglobin (Hb), Hematocrit (Hct), Red Blood Cells (RBCs) and RBC indices, Red Cell Distribution Width (RDW), platelets, serum iron, Transferrin, percent transferrin saturation (%Tsat) defined as serum iron divided by serum transferrin multiplied by 100%, serum ferritin, albumin, SGOT, SGPT, serum creatinine. Several definitions for ID were suggested [23] and here we defined ID by three different criteria: (i) serum ferritin of <100 ng/ml, (ii) %TSat <20%, or (iii) either a serum ferritin of <100 ng/ml or the combination of serum ferritin of 100-300 ng/ml and %TSat of <20%. Estimated glomerular filtration rate (eGFR) was calculated by the MDRD equation [24].

Pulmonary function was performed by spirometry which was performed in accordance with the recommendations of the American Thoracic Society [25]. The one second Forced Expiratory Volume (FEV 1), Forced Vital Capacity (FVC) and the FEV1/FVC, all assessed as percent of predicted values, were measured before but not after the EPO-IV iron therapy.

Health perception was assessed at baseline and one week after the fifth injection of EPO and IV iron. We used a Visual Analog Scale (VAS) making a line 10 cm in length, in which a score of 0 at one end corresponds to extremely severe dyspnea on walking a short distance or at rest and a score of 10 at the other end corresponds to no dyspnea on walking or at rest. The patient placed an X on what they considered their current status before and 1 week after the last dose of the combination treatment.

### Statistical analysis

All results of *Study 2* are reported as median and interquartile range [IQR]. All variables did not follow a normal distribution and were therefore analyzed by the Wilcoxon signed rank sum test to evaluate the changes in them after treatment. The Spearman correlation coefficients were calculated between pre- and post values of all parameters to examine the relationships between the two measurements. A two-tailed value of p < 0.05 was considered to be significant.

Statistical analysis was performed by SAS for windows version 9.2.

## Results

### Study 1- Prevalence of anemia and iron deficiency in patients hospitalized with AECOPD

Of 107 consecutive hospital records examined of patients admitted with AECOPD, 47 patients (43.9%) were found to be anemic on admission (Hb < 12 g/dl) and 60 (56.1%) not anemic (Table 1). Only two (3.3%) of the 60 non-anemic patients were investigated for ID (serum iron, %TSat and serum ferritin) as compared to18 (38.3%) of the 47 anemic patients. All 18 (100%) of the anemic patients had ID by our criteria, yet none had oral or IV iron subscribed either before admission, during hospitalization, or at the time of discharge. In the 18 anemic patients, if ID was only defined as a serum ferritin of <100 ng/ml it was found in 11/18 (61.1%). If ID was defined only as %TSat <20%, in 18/18 (100%). If defined as either a serum ferritin of <100 ng/ml or the combination of serum ferritin of 100-300 ng/ml and%TSat of <20% it was found in 18/18 (100%). By whatever definition used, ID was very common. In the two non anemic patients, in which the iron status was measured, one had a %TSat of 40% and a serum ferritin of 200 ng/ml and was not iron deficient while the other had a %TSat of 14% and a serum ferritin of 160 ng/ml and was iron deficient.

**Table 1:** Anthropological and clinical characteristics of 107 patients hospitalized with an acute exacerbation of COPD

**Characteristics**	**Non-anemic patients (n = 60)**	**Anemic patients (n = 47)**
Age (years; mean + SD)*	67.1 ± 11.2	77.6 ± 9.5
Sex (male/female)	33/27	19/28
**Hematological parameters**		
Hb (mg/dL; mean ± SD)	14.2 ± 1.4	10.6 ± 1.3
Hct (%; mean ± SD)	42.8 ± 4.6	32.5 ± 4.1
MCV (fl; mean ± SD)	86.8 ± 10.7	86.0 ± 9.2
RBC (10^6^/μl; mean ± SD)	4.8 ± 0.6	3.7 ± 0.5
MCH (pg; mean ± SD)	31.3 ± 9.2	28.7 ± 2.6
MCHC( g/dl; mean ± SD)	33.0 ± 1.5	32.5 ± 1.0
**Co-morbidities**		
CHF (%)	10 (17%)	14 (30%)
CHD (%)	13 (22%)	9 (19%)
Hypertension (%)	21 (35%)	17 (36%)
Diabetes Mellitus (%)	11 (18%)	15 (32%)
Atrial Fibrillation (%)	4 (7%)	10 (21%)
Peripheral Vascular Disease	4(7%)	8 (17%)
Creatinine ≥ 1.5 mg/dl (%)	11 (18%)	17 (36%)

*Abbreviations*: CHF, congestive heart failure; CHD, coronary heart disease; Hb, hemoglobin; Hct, hematocrrit; SD, standard deviation; *, p > 0.05 (not statistically significant).

### Study 2- Outpatients intervention study

#### *Baseline assessment*

The anthropological and clinical characteristics of the 12 patients (Table 2) showed a median age of 74.0 years [66.5-79.5]. A past medical history of hypertension, CKD, CHF, diabetes and dyslipidemia and a past or present history of smoking were very common; indeed all had CKD. Angiotensin Converting Enzyme Inhibitors (ACEIs) and/or Angiotensin II Receptor Blockers (ARBs), beta blockers and statins were being used frequently. All were on bronchodilators.

**Table 2:** Anthropological and clinical characteristics of 12 outpatients with COPD, treated with ESAs and intravenous iron for anemia

**Characteristics**	**Number (%)**
**Age (years)**	74.0 [66.5–79.5]
**Male**	6 (50%)
**FEV1 (% predicted)**	46.5% [40–55]
**FEV1/FVC (% predicted)**	65.5 [49–68]
**Basal Oxygan saturation (%)**	94.5 [92–96]
**CHF**	9 (75%)
**Diabetes Mellitus**	6 (50%)
**Hypertension**	11 (92%)
**CKD**	12 (100%)
**Dyslipidemia**	8 (67%)
**Obesity**	3 (25%)
**Smoking**	8 (67%)
**Inhaled Bronchodilators**	12 (100%)
**ACE-I**	7 (59%)
**ARBs**	3 (25%)
**Beta-blockers**	10 (84%)
**Statins**	8 (67%)

*Abbreviations*: ACE-I, angiotensin converting enzyme inhibitors; ARBs, angiotensin receptor ii blockers; CHF, congestive heart failure, CKD, chronic kidney disease; ESAs, erythropoiesis stimulating agents; FEV1, one second Forced Expiratory Volume; FVC, Forced Vital Capacity.

The baseline FEV1 was 46.5% [40–55] of predicted values and the baseline FEV1/FVC was 65.5% [49–68] of predicted values. The median basal oxygen saturation measured by pulse oximetry was 94.5% [92–96].

### Red cell indices

The anemia was normocytic and normochromic in all, with normal mean values for MCH, MCV and MCHC. The mean RDW was increased.

### Prevalence of iron deficiency

If defined as only a serum ferritin of <100 ng/ml it was found in 6/12 (50%). If defined only as %TSat <20%, in 9/12 (75%). If defined as the combination of serum ferritin of <300 ng/ml and %TSat of <20% it was found in 9/12 (75%). If defined (as we defined it) as either a serum ferritin of < 100 ng/ml or the combination of serum ferritin of 100-300 ng/ml and %TSat of <20%, it was found in 11/12 (91.6%) of the cases. By whatever definition used, iron deficiency was very common in these ambulatory anemic COPD patients.

### Results of treatment

#### *Hemoglobin and red cell indices*

The Hb rose from an initial median of 9.9 g/dl [9.2-10.6] to 12.35 g/dl [11.6-13.0] (p = 0.0005) by one week after the last injection. The Hct similarly increased from 29.9% [28.0-31.5] to 38.1% [35.0-39.4] (p = 0.0005). The median RBC count increased from 3.3 cells/mcL [3.2-3.7] to 3.9 cells/mcL [3.6-4.3] (p = 0.01).

### Iron indices

The serum ferritin and %TSat increased significantly by one week after the last dose versus baseline. Serum ferritin from 99 ng/ml [48.6-127.4] to 330.6 ng/ml [243.4-615.2] (p = 0.005) and %TSat from 12.8% [11–19.1] to 24.0% [18.5-27.9] (p = 0.005).

### Other blood parameters

The changes in the RDW, WBC, lymphocytes and platelets were not significant (Table 3).

**Table 3:** Biochemical and hematological parameters of 12 outpatients with COPD, before and after treatment with ESAs and intravenous iron

**Characteristics**	**Baseline**	**After treatment**	**p**
Weight kg	77.2 [69.2–90.5]	77.9 [70.2–88.1]	0.32
DBP mmHg	67.5 [60–88]	70.1 [66–85]	0.19
SBP mmHg	138 [ 120–158]	134 [121–156]	0.05
Hb g/dl [11.7–15.5]	9.9 [9.2–10.6]	12.4 [11.6–13]	0.0005
Hct% [35–45]	29.9 [28–31.5]	38.1 [35.0–39.4]	0.0005
MCV fl [80–96]	89.0[85–92]	90 [87–92]	0.17
RBC 10^6^/μl 4.3–5.8	3.3 [3.2–3.7]	3.9 [3.6–4.3]	0.01
MCH pg [26–34]	30.5 [29.3–31.7]	29.8 [29.0–31.6]	0.99
MCHC g/dl [31–37]	33.3 [32.6–34.2]	33.4 [32.9–34.3]	0.8
RDW%	14.9 [13.1–16.5]	15.4 [14.6–16.5]	0.13
WBC`10^3^/ul [4–11]	8.0 [4.9–9.2]	7.1 [6.0–8.3]	0.38
Lymphocytes% WBC [20–40]	19.2 [16.1–21.0]	21.0 [18.0–23.9]	0.6
Platelets 10^3^/μl [150–450]	237 [193–303]	220 [170–282]	0.51
TSAT% [20–55]	12.8 [11.0–19.1]	24.0 [18.5–27.9]	0.005
Ferritin ng/ml [220–400]	99 [48.6–127.4]	330.6 [243.4–615.2]	0.005
Iron mcg/dl [40–150]	44,5 [29–61]	61.5 [57–74]	0.015
Serum creatinine mg/dl [0.7–1.3]	2.4 [1.8–3.6]	2.4 [1.9–3.5]	0.21
eGFR ml/min/1.73 m^2^ [ > 90]	27.5 [19–37]	28.50 [21–39]	0.56
GOT U/l [7–40]	20 [17–39]	26 [15–41]	0.04
GPT U/l [5–39]	16 [11–41]	21 [10–40]	0.04
Albumin g/l [35–50]	38.5 [36–42]	39.0 [35–41]	0.36
VAS [0–10]	2.5 [2–3]	8.5 [7–10]	0.0005

Values are presented as median with interquartile range [IQR] and reference intervals.

*Abbreviations*: DBP, diastolic blood pressure; eGFR, estimate glomerular filtration rate; ESAs, erythropoiesis stimulating agents; GOT, alanine amino-transferase; GPT, aspartate amino-transferase; Hb, hemoglobin; Hct, hematocrit; MCV, mean corpuscular volume; MCH, mean corpuscular hemoglobin; MCHC, mean corpuscular hemoglobin concentration; RBC, red blood cell count; RDW, red cell distribution width; SBP, systolic blood pressure; VAS, Visual Analogue Scale; WBC, white blood cell count; %TSat, % transferrin saturation.

### Hemoglobin and Hematocrit correction and improvement of dyspnea

The VAS scale increased from an initial 2.5 [2–3] to 8.5 [7–10] (p = 0.0005).

**Figure 1:**
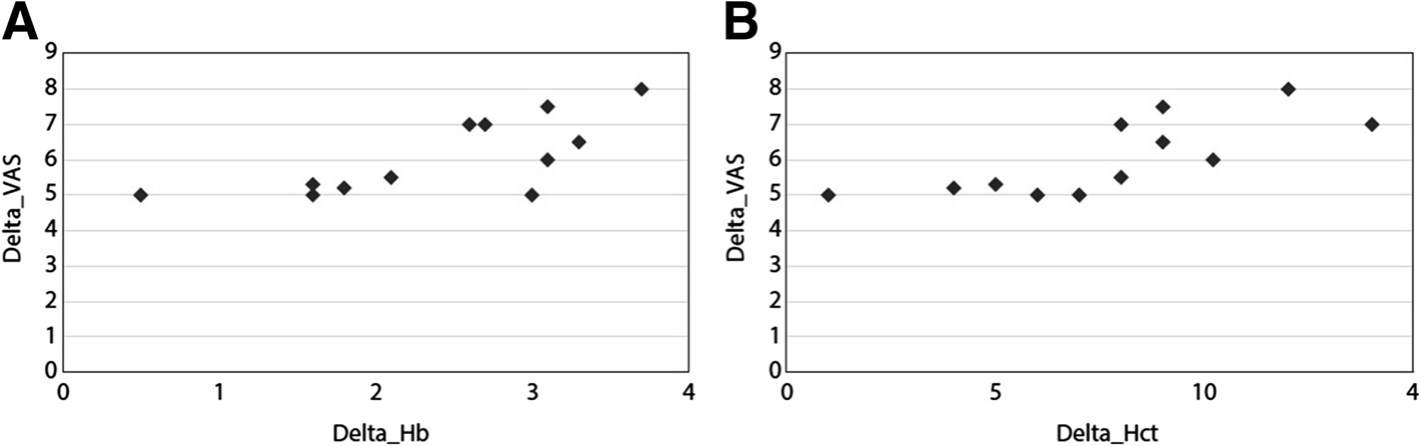
**Correlation between anemia parameters and the Visual Analog Scale (VAS) score in 12 COPD patients treated with ESAs and intravenous iron. A**. Correlation between the delta hemoglobin (Hb) and delta VAS. **B**. Correlation between the delta hematocrit (Hct) and delta VAS.

There was no significant correlation between baseline Hb and VAS or baseline Hct and VAS. There was a highly significant correlation between the ∆Hb and ∆VAS; r_s_ = 0.71 p = 0.009 and between the ∆Hct and ∆VAS; r_s_ = 0.8 p = 0.0014 (Figure 1).

### Other effects

As seen in Table 3 the mean SBP and DBP, weight, BMI, serum albumin, cholesterol, SGOT, and SGPT remained unchanged. Neither the serum creatinine nor the eGFR changed significantly.

### Clinical follow-up

The median follow-up period after termination of ESAs and IV-iron treatment was 1.9 years (range 0.4-4.5 years). Four patients died after follow-up of 0.4 to 3.8 years. Of these four patients, two reached end-stage renal disease with the need for hemodialysis. Only one of the four deaths was directly attributed to respiratory disease. Six patients continued treatment in our clinic with ESAs and/or IV iron therapy as needed and another two patients were lost during follow-up.

AECOPD episodes, in the first year after anemia treatment, were documented in seven of the nine patients that were followed for more than a year. The median time from termination of anemia therapy to first exacerbation in these seven patients was 131 days (range 23–307 days).

## Discussion

We have shown that anemia and ID are very common in hospitalized AECOPD patients but ID is neither sought for or, if found, is not treated. Patients hospitalized for AECOPD have relatively severe COPD. It was suggested that the prevalence of anemia is likely higher in this group compared to the general COPD population [26-28]. Surprisingly, there are scarce data about ID in COPD. Recently, Comeche Casanova *et al.* have shown in a cohort of 130 patients with stable COPD that serum iron was significantly lower in anemic patients than in those without anemia [29].

We have also shown in a small retrospective study in anemic outpatients with moderate to severe COPD that treatment with ESA and IV-iron significantly improved the anemia and ID, and that this was associated with a significant improvement in self-assessed shortness of breath. The improvement in the VAS dyspnea score was directly related to the increase in Hb and Hct. The hematological improvement of the anemia in this group of patients with COPD was similar to the effect that we have previously shown when ESAs and IV-iron were used in renal failure and heart failure, even without COPD [21,22].

These results are consistent with the findings of others who treated anemia in COPD with either anabolic steroids [30] or blood transfusions [30,31]. The study involving the use of anabolic steroids in COPD [30] showed that the improvement in the Hb that was found was associated with an improvement in pulmonary function as judged by maximal inspiratory mouth pressure and peak workload. In another study, transfusion of a mean of 2.2 units of packed cells in 10 anemic COPD patients in an intensive care unit was shown to lead to a significant reduction of both minute ventilation and work of breathing [31]. In a second study by the same group [32], blood transfusions allowed five anemic COPD patients, in whom trials of weaning from a respirator before the transfusions had been unsuccessful, to be successfully weaned after the transfusions. All these studies, in addition to the present study, suggest that correction of the anemia in COPD may improve pulmonary functions and respiratory symptoms.

Anemia in patients with COPD is likely due to a combination of several factors [26,29,33]: (i) Elevated cytokines levels, especially Tumor Necrosis Factor alpha (TNF α) and interleukin-6 (IL-6) [1,12,17,18,34,35]. Moreover, persistent inflammation may be associated with poor clinical outcome for COPD patients [36]. The elevation of cytokines can lead to reduced production of erythropoietin (EPO), reduced erythropoietic response of the bone marrow to EPO (i.e. resistance to EPO), hepcidin-induced failure of iron absorption from the gut, and hepcidin-induced trapping of iron in iron stores in the macrophages and hepatocytes [33]. (ii) Concomitant CKD with reduced production EPO [37]. (iii) Use of Angiotensin Converting Enzyme Inhibitors (ACE-I) and Angiotensin Receptor Blockers (ARBs) that can cause reduced activity of EPO in the bone marrow [38]. (iv) Concomitant Diabetes Mellitus [39] and (v) Gastrointestinal causes, especially with use of steroids [32].

There have been several studies suggesting that ID may contribute to pulmonary dysfunction. In a recent US Third National Health and Nutrition Examination survey (NHANES) [20] there was a direct correlation between pulmonary function as judged by the one second forced expiratory volume (FEV1) and the iron status as judged by the %TSat and ferritin, suggesting that reduced pulmonary function may be commonly associated with ID. In addition ID is common in cystic fibrosis [40] and pulmonary tuberculosis [41]. Pulmonary hypertension of different causes is often associated with iron deficiency and indeed treatment of iron deficiency can improve pulmonary hypertension [42-45].

In CHF, the correction of the anemia with ESAs and oral or IV iron or even with IV iron alone has been shown in many studies to improve cardiac and patient function and cardiac structure [33,46-49], and more than 20% of COPD patients have also CHF [50]. However,

a recent large long-term double blind study with the ESA, Darbepoetin alpha, did not meet its primary endpoint of reducing the composite endpoint of time to death from any cause or first hospital admission for worsening heart failure in patients with systolic CHF [51,52]. Thus the role of ESAs alone in CHF is still uncertain. There are concerns about complications related to ESAs therapy, since its use in renal failure has been associated with an increase in cardiovascular and thromboembolic phenomena, especially when given in high doses [53-57]. Correction of ID with or without anemia in CHF may improve symptoms, functional capacity, and quality of life [58]. However, some concerns have also been raised about the safety of IV iron with its possible worsening of oxidative stress and reduced immunological function with possible increased tendency to infections [59,60].

Our study has limitations as it is a non-randomized, retrospective observation in a single medical center. In the intervention study (***study 2***), the patients had a very high rate of comorbidities including CKD, CHF, diabetes and hypertension- a higher rate than is seen with the usual COPD patients. In fact, all 12 patients had CKD and it is possible that CKD predominated over their other comorbidities. However the doses of CKD and CHF medications were unchanged during the study. Unfortunately we did not study pulmonary function at the termination of the treatment period in any of these 12 patients and are therefore unable to assess the effect of anemia correction on lung function. Another limitation is that the VAS scale for dyspnea was used since it is the common practice in our clinic. For patients with COPD it is preferred to use the Modified Medical Research Council Dyspnea Scale (mMRC) [61]. Nevertheless, our results here suggest that correction of anemia with ESAs or iron may benefit COPD patients, similarly to the benefits seen in CHF.

Any studies of anemia in COPD using ESAs, IV Iron or the combination of these agents, should be adequately powered, double-blind placebo-controlled, and of sufficient duration to assess the advantages and disadvantages of the therapy. But such an effort would seem to us worthwhile in these anemic and frequently iron deficient COPD patients in view of the possible improvement in cardiac and pulmonary function, hospitalization and mortality, exercise capacity and Quality of Life that might occur with correction of the their anemia and/or iron deficiency.

## Conclusions

These studies suggest that ID is common in hospitalized COPD but generally not sought after and, if found, generally not treated. Our small retrospective intervention outpatient study also suggested that correction of anemia and ID in COPD patients may improve dyspnea. If further studies confirm our preliminary observations, evaluation and treatment of ID could become standard practice in COPD as is currently the case for CKD [62] and CHF [63].

## Consent

Written informed consent was obtained from the patient for the publication of this report and any accompanying images.

## Competing interests

The authors declare that they have no competing interests.

## Authors’ contributions

DSS- participated in the design and draft of manuscript (the major contributor). RM- participated in the acquisition and analyzing of clinical data and in the draft of manuscript. MTW,EB,DS,IFS- participated in the acquisition and analyzing of clinical data. GC- conceived of the study, and participated in its design and critically revisited the draft. All authors read and approved the final manuscript.

## Pre-publication history

The pre-publication history for this paper can be accessed here:

http://www.biomedcentral.com/1471-2466/14/24/prepub
